# The effect of leaf hydroalcoholic extract of Ephedra pachyclada infertility in male rats treated with cyclophosphamide: An experimental study

**DOI:** 10.18502/ijrm.v21i4.13268

**Published:** 2023-05-08

**Authors:** Shiva Saleh, Aref Ghanaatpisheh, Hoda Haghshenas, Negar Parvin, Elmira Mikaeiliagah, Hossein Kargar Jahromi, Bahare Ebrahimi

**Affiliations:** ^1^Student Research Committee, Jahrom University of Medical Sciences, Jahrom, Iran.; ^2^Stem Cell and Tissue Engineering Laboratory, Department of Orthopedics, West Virginia University, Morgantown, USA.; ^3^Department of Biology, Ardabil Branch, Islamic Azad University, Ardabil, Iran.; ^4^Research Center for Noncommunicable Diseases, Jahrom University of Medical Sciences, Jahrom, Iran.; ^5^Shiraz Geriatric Research Center, Shiraz University of Medical Sciences, Shiraz, Iran.

**Keywords:** Ephedra, Cyclophosphamide, Cancer, Spermatogenesis, Sex hormones, ROS

## Abstract

**Background:**

Cyclophosphamide (CP) has clinical applications in treating diverse malignancies and autoimmune disorders; at the same time, it also has harmful effects on the body tissues, particularly the genitals. The most significant side effects of CP are changing the reproductive system's function and infertility.

**Objective:**

This study determines the Ephedra hydroalcoholic extract (EP) role on testicular tissue and the pituitary-gonadal axis in CP-treated male rats.

**Materials and Methods:**

In this experimental study, 48 adult Wistar rats were separated into 6 groups (n = 8/each): control, sham, CP recipients, and CP recipients with gavage-fed EP (250, 500, and 1000 mg/kg). On the 29 ${}^{\text{th}}$ day, the blood of the weighed animals' was drawn from their heart, and serum concentrations of follicle-stimulating hormone, luteinizing hormone, and testosterone were measured. After preparing testicular tissue segments, cells were counted.

**Results:**

While CP decreased follicle-stimulating hormone, luteinizing hormone, and testosterone levels (p < 0.05), the use of EP changed them and even reached the control. Serum gonadotropin-releasing hormone increased significantly in all EP groups compared to the control and CP groups. Compared to the control, a significant decrease in total antioxidant capacity and plasma glutathione peroxidase was observed in the CP groups. EP (all doses) significantly increased their concentration compared to the CP group (p < 0.05); significant reduction in serum total oxidant status and malondialdehyde in CP groups changed by EP (p < 0.05). Although CP's role on spermatogonia counts (57.5 ± 5.2 in CP, 67.1 ± 6.0 in control), higher doses of EP had no significant effect on this but did affect spermatocyte and spermatid cells count.

**Conclusion:**

Due to its antioxidant characteristics, EP mitigated the effects of CP on the investigated parameters in rats.

## 1. Introduction

Cancer is known to be the second cause of mortality in over 100 countries, affecting life expectancy and patient's quality of life. From the past decades till now, rational use of effective anti-cancer agents for treatment was felted (1); among all discovered drugs, cyclophosphamide (CP), a nitrogen mustard anti-cancer agent, has been consumed as a chemotherapeutic drug for more than 50 yr after its discovery (2-3). Despite CP's clinical benefits, some significant adverse effects of CP, such as nephrotoxicity (4), cardiotoxicity (3), severe bladder bleeding, and gonadotoxicity had limited its clinical indication (5-7). In the presence of cytochrome P450, CP metabolizes into 2 toxic compounds, acrolein, and phosphoramide. Acrolein is an unsaturated and electrophilic reactive aldehyde that generates noxious reactive oxygen species (ROS) and next, affects the neighboring tissue (8, 9). Studies have defined the acrolein-induced excessive ROS effects on testicular microvascular, endocrine signaling, and germ-cell apoptosis which causes testicular infertility (10). Besides them, CP increases the incidence of early menopause and infertility in males and females; this type of infertility is often transitory, but it can potentially be permanent (11, 12). In a study by Watcho et al., CP-treated Wistar rats had significant reduction in seminal vesicle weight, testosterone level, sperm count, motility, and viability; furthermore, sperm morphological abnormality was also detected (13). In addition to morphological and qualitative changes in sperm caused by CP, there are also studies that have shown DNA damage and nuclear maturations (14).

In the last few years, considerable interest in the use of medicinal plants with the aim of cancer treatment or reducing anticancer agents' side effects has been increased. CP applies its effect primarily by persuading oxidative stress and changing gene expression in spermatocytes vastly unlike stages of development (15); therefore, these studies mostly rely on the antioxidant role of plants and consider antioxidants as affective factors (13, 14). For example, in a study by Can et al., useful Hypericum Triquetrifolium Turra against CP gonadotoxicity had elevated total oxidant status (TOS), and malondialdehyde (MDA) in the CP group (16).

Ephedra (EP) is a major genus of the Ephedraceae family, found in dry and waterless regions of the globe. Some members of this genus are extensively used in traditional medicine to ameliorate and regulate asthma's symptoms, common cold, influenza, fever, and headache (17). Due to ephedrine-type alkaloids and their pharmacological characteristics, the chemical ingredients of EP species have been the subject of investigation for decades (10). Its role in cardiovascular and central nervous systems, nephrotoxicity and hepatotoxicity, and cancers such as breast cancer were examined in different studies (18-20). Despite the known antioxidative function of EP (21, 22), in only one in vivo study, EP had significantly bring back testicular weight and activity, spermatozoid characteristics, serum and tissue hormonal levels, and antioxidant protection. This study had shown significantly activated gonadotropin-releasing hormone (GnRH) signaling pathway and meiosis-related protein levels by EP in rat model of adriamycin injury (23).

Considering today's high prevalence of cancer and CP role as an essential anticancer drug, special attention should be paid to this drug's side effects. To compensate for the side effects of these chemotheraputic drugs, herbs, especially those with antioxidative function would be useful. We turned to using EP, a plant with proven antioxidative functions. This study sought to determine EP hydroalcoholic extract role on testicular tissue and the pituitary-gonadal axis in CP-treated male rats.

## 2. Materials and Methods

### EP collection and extraction

EP was collected from Yasuj, Iran, and then the plant's stems were dried at room temperature and powdered. EP powder was mixed with ethanol (70%) solution at a chamber temperature (35 ± 5 C) and after 4 days “the extract was filtered using a sterile cloth sheet. The extract was dried under reduced pressure at below 45 C with a rotary evaporator. This extract were stored at 4 C” (24).

### Determination of extract purification

At first, 1 ml of phenol solution was combined with 200 µl EP pachyclada extract and “stored in dark for 6 min, then 2 ml of NaCo ${}_{3}$ (7%) was added and kept for 120 min. The solution absorbance was measured at a wavelength of 765 ” and finally, the total phenolic amount of extract was calculated using the standard curve of gallic acid and reported 9.28 mg/(GAE)4/g (25). Total flavonoid measurement was calculated by the method of Dharmadasa et al. and 44.32 mg of (QE) 5/g extract was reported based on the quercetin standard curve (26).

### Experiment design

In this experimental study, 48 Wistar male rats (180-200 g) were divided into 6 groups. The animals were kept in the animal laboratory of Jahrom University of Medical Sciences, Jahrom, Iran with standard laboratory conditions of 12 hr light/dark cycles and 50-55% of humidity.

According to the previous studies, EP extract was prescribed with concentrations of 250, 500, and 1000 mg/kg by gavage (24). The CP (Baxter, Germany) concentration was determined 5 mg/kg (for 28 days) (27); the control group did not receive any treatment during the experiment, and the sham group received distilled water and alcohol-based on body weight by gavage. Experimental group 1 received only 5 mg/kg CP, and experimental groups received CP and EP extract 250, 500, and 1000 mg/kg.

### Biochemical analysis

On the 29 ${}^{\text{th}}$ day, the blood of the weighed animals' was drawn from their heart (under anesthesia using Xylazine (10 mg/kg of body weight) and Ketamine (50 mg/kg of body weight)). Their serums were collected via centrifugation (for 15 min and 3000 rpm) and then kept at -20 C until laboratory analysis. To measure the luteinizing hormone (LH), follicle-stimulating hormone (FSH), testosterone, GnRH, TOS, MDA, total antioxidant capacity (TAC), and plasma glutathione peroxidase (GSH-PX), the ELIZA kits for rats were used (Shanghai Crystal day Biotech Co., China).

### Histological examination 

For histological examination, testis tissue (left and right) was rapidly removed and placed in a 10% formalin container. After 48 hr, the testis tissue was removed from the solution and after routine tissue processing; paraffin sections of 5 μm thickness were prepared as serial sections. Sections of each testis were stained by the H&E method. The mean number of spermatogonia, spermatocytes, spermatids, Leydig, and Sertoli cells were assessed in the testis by stereology method. To count the cells, the optical dissector method and unbiased counting frame were used. The counting frame was randomly placed on the fields of view selected from each slice (10,000 × magnification), from each side (top and bottom) up to a depth of 5 µ, sections with artifacts were considered, and counting was not done in those sections. Cells selected by the frame were counted at other depths (average 130-150 of each cell). The desired depths were obtained with a microcator (MT12, Germany). Then, the numerical density of the cells was obtained and multiplied by the final volume of the testis, in order to achieve the estimated cell count.

### Ethical considerations

This study was in agreement with ethical issues of animal laboratory Ethics of Jahrom University of Medical Science, Jahrom, Iran (Code: IR.JUMS.REC.1396.018).

### Statistical analysis

SPSS software (version 24) was used for data analysis. At first, the normality of the data was measured using Shapiro-Wilk normality test. Data were analyzed by Independent-samples *t* test using SPSS software version 16 and the significance level was considered 0.05.

## 3. Results

### Changes in serum LH, FSH, testosterone, and GnRH

Data analysis showed that CP had affected serum FSH and LH and using EP had altered these; using higher doses of EP (500 and 1000 mg/kg) were not significantly different with the control. All experimental groups showed a significant decrease in serum testosterone compared to the control group at p < 0.05. The mean testosterone concentration in CP and EP 500 mg/kg did not significantly differ from the CP group; however, the higher doses were significantly different from CP group and the best effect belonged to the highest dose.

Serum GnRH concentration showed a significant increase in all EP groups compared to the control and CP groups. The mean serum concentration of GnRH in experimental groups containing 500 and 1000 mg/kg EP was significantly higher than the group treated with 250 mg/kg EP (p < 0.05).

On comparing CP groups with EP extract ones, it was found that 1000 mg/kg EP had more significant effects on boosting serum-assessed parameters (Table I).

### Changes in serum antioxidants, MDA, and TOS

Data analysis showed a significant reduction of serum TAC and GSH-PX in groups that used CP compared to the control. EP (all doses) significantly increased TAC concentration compared to the CP group (p < 0.05); however, GSH-PX data showed this significant increase only in higher doses of EP.

A significant difference was observed in serum sets of TOS and MDA in all experimental groups compared to the control group. In addition, serum levels of TOS and MDA in EP groups compared to the CP group showed a significant reduction at the p < 0.05 (Table II). Each group means with at least one shared letter had no significant difference.

**Figure 1:**
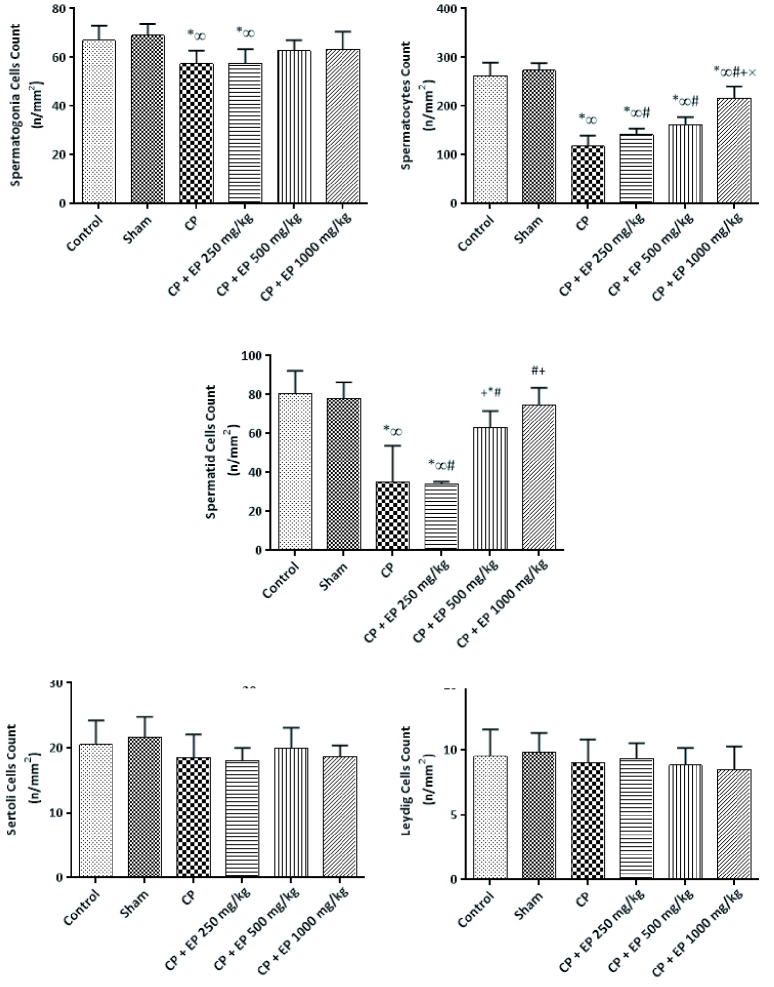
The effect of cyclophosphamide (CP) and Ephedra (EP) on male Wistar rats spermatogonia, spermatocytes, spermatid, Leydig, and Sertoli cell. Each group means with at least one shared letter has no significant difference. P < 0.05 is considered statistically significant, Independent-samples *t* test.

Changes in the mean number of spermatogonia, spermatocytes, spermatid, Leydig, and Sertoli cells:

This study showed that despite the role of CP on the mean number of spermatogonia (p < 0.05), experimental groups, received higher doses of EP with no significant difference with the control group. No significant difference was observed between the CP groups and the 250 mg/kg EP group. All experimental groups showed a significant decrease in spermatocyte count compared to the control group at p < 0.05, but using EP affected this significantly compared to the CP group. Moreover, no significant difference was observed between 500 and 1000 mg/kg EP. The mean changes of spermatid cells in CP groups except the group treated with 1000 mg/kg EP was significantly lower than the control group. Higher doses significantly increased the spermatid cells count compared to the CP and CP+250 mg/kg EP groups. The mean number of Leydig and Sertoli cells in all study groups did not significantly differ compared to the control group and EP did not affect them (Figure 1).

### Testicular tissue histological results

**Figure 2:**
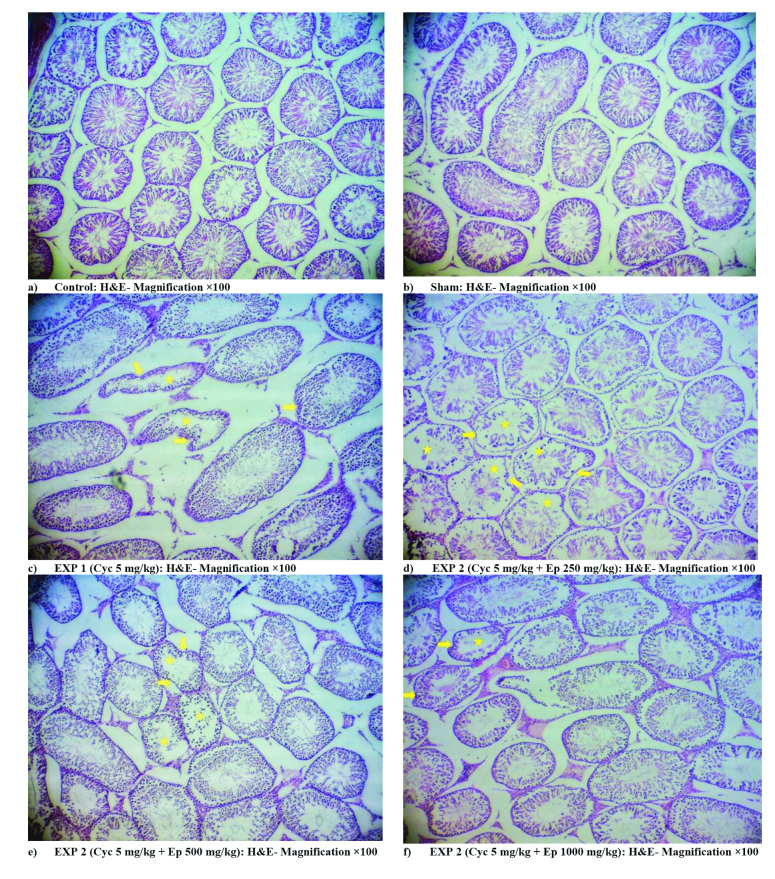
The effect of 5 mg/kg cyclophosphamide and different doses of Ephedra (EP) on male Wistar rats' testicular tissue. Arrows: Germinal epithelium of seminiferous tubule, Stars, Lumen of seminiferous tubule.

The histological examination of the testicular tissue showed that no tissue changes were observed in the control and sham groups. In the CP group, cell disorganization and disorders inside the seminiferous tubule, and wrinkling of the germinal epithelium were observed. In groups receiving EP with different doses, tissue disorders showed less severity than the CP group receiving CP. By increasing the concentration of EP, the intensity of these changes decreased so the lowest amount of tissue disorders was observed in the EP 1000 mg/kg group (Figure 2).

**Table 1:** The effect of CP and EP on male Wistar rats FSH, LH, Testosterone, and GnRH


**Variables **	**FSH (mIU/ml)**	**LH (mIU/ml)**	**Testosterone**	**GnRH (ng/L)**
**Control**	1.613 ± 0.06	1.565 ± 0.02	2.197 ± 0.18	807.7 ± 18.5
**Sham**	1.609 ± 0.02	1.547 ± 0.01	2.210 ± 0.09	801.1 ± 29.9
**CP**	1.321 ± 0.12 ${}^{*\infty }$	1.385 ± 0.06 ${}^{*\infty }$	1.345 ± 0.01 ${}^{*\infty }$	526.517 ± 37.3 ${}^{*\infty }$
**CP + Ep 250 mg/kg**	1.353 ± 0.04 ${}^{*\infty \#}$	1.412 ± 0.03 ${}^{*\infty \#}$	1.445 ± 0.08 ${}^{*\infty \#}$	604.633 ± 52.7 ${}^{*\infty \#}$
**CP + Ep 500 mg/kg**	1.534 ± 0.09 ** ${}^{\#+}$ **	1.596 ± 0.04 ** ${}^{\#+}$ **	1.650 ± 0.11 ${}^{*\infty \#+}$	693.35 ± 41.7 ${}^{*\infty \#+}$
**CP + Ep 1000 mg/kg**	1.637 ± 0.09 ** ${}^{\#+}$ **	1.538 ± 0.03 ** ${}^{\#+}$ **	1.912 ± 0.16 ${}^{*\infty \#+\times }$	735.65 ± 26.5 ${}^{*\infty \#+}$
**P-value (between groups)**	< 0.001	> 0.01	< 0.001	< 0.001
Data presented as Mean ± SD. Independent-samples *t* test. *Difference with control, ** ∞ ** Difference with Sham, **#**Difference with CP, **+**Difference with CP + Ep 250 mg/kg, ** × ** Difference with CP + Ep 500 mg/kg, CP: Cyclophosphamide, EP: Ephedra hydroalcoholic extract, FSH: Follicle-stimulating hormone, LH: Luteinizing hormone, GnRH: Gonadotropin hormone-releasing hormone				

**Table 2:** The effect of CP and EP on male Wistar rats serum antioxidants, MDA, and TOS


**Variables **	**GSH-PX (U/ml)**	**TAC (U/ml)**	**TOS (U/ml)**	**MDA (nmol/ml)**
**Control**	1476.33 ± 96.5	2.05 ± 0.4	0.044 ± 0.00	1.37 ± 0.1
**Sham**	1444.11 ± 67.2	2.03 ± 0.3	0.042 ± 0.01	1.32 ± 0.2
**CP **	212.81 ± 31.0 ${}^{*\infty }$	0.58 ± 0.1 ${}^{*\infty }$	0.197 ± 0.01 ${}^{*\infty }$	7.09 ± 0.7 ${}^{*\infty }$
**CP + Ep 250 mg/kg**	251.06 ± 13.5 ${}^{*\infty \#}$	0.92 ± 0.1 ${}^{*\infty \#}$	0.159 ± 0.00 ${}^{*\infty \#}$	4.35 ± 0.5 ${}^{*\infty \#}$
**CP + Ep 500 mg/kg**	791.86 ± 45.6 ${}^{*\infty \#+}$	1.34 ± 0.1 ${}^{*\infty \#+}$	0.145 ± 0.01 ${}^{*\infty \#+}$	2.69 ± 0.4 ${}^{*\infty \#+}$
**CP + Ep 1000 mg/kg**	1203.93 ± 96.5 ${}^{*\infty \#+\times }$	1.43 ± 0.1 ${}^{*\infty \#+}$	0.094 ± 0.01 ${}^{*\infty \#+\times }$	1.94 ± 0.2 ${}^{*\infty \#+\times }$
**P-value (between groups)**	< 0.001	< 0.001	< 0.001	< 0.001
Data presented as Mean ± SD. Independent-samples *t* test. *Difference with control, ** ∞ ** Difference with Sham, **#**Difference with CP, **+**Difference with CP + Ep 250 mg/kg, ** × ** Difference with CP + Ep 500 mg/kg. CP: Cyclophosphamide, EP: Ephedra hydroalcoholic extract, GSH-Px: Plasma glutathione peroxidase, TOS: Total oxidant status, TAC: Total antioxidant capacity, MDA: Malondialdehyde				

## 4. Discussion

Gonadotoxicity has become one of the most notable and commonly encountered side effects of extended CP use, so there is an imperative and crucial need to identify therapeutic strategies to decrease or regulate the reproductive potential of cancer patients subjected to CP therapy (28). CP is metabolized by cytochrome P450 to 2 stable toxic composites: Acrolein, an α, β-unsaturated aldehyde that has an extremely electrophilic feature, and phosphoramide mustard. ROS mainly induces Acrolein, and Acrolein, induces ROS and oxidative damage via DNA cross-linking and interfering in rapidly proliferating organs and tissues such as testicular tissue, which is known well (29, 30). For example, in a study, it was shown that CP induced gonadotoxicity would be affected by using Nerolidol, a sesquiterpene with potent antioxidative properties. Nerolidol acted as a gonadoprotective molecule and increased testosterone level, sperm count, as well as it also reduced MPO (myeloperoxidase) expression and improved CP-induced histological abnormalities in testes, seminal vesicles, and even epididymis (7). Different studies had shown the beneficial effect of herbs on CP-induced tissue damages, such as gonadotoxicity (31, 32).

This study revealed the gonadoprotective effects of the leaf hydroalcoholic extract of EP pachyclada counter to gonadotoxicity caused by CP in male rats by indicating its effect on serum concentration of FSH, testosterone, and LH. The results showed that the hydroalcoholic extract of EP significantly increased the mean serum levels of FSH, LH, and testosterone; EP also affected testicular spermatocytes, spermatozoids, and spermatids in a dose-dependent manner, especially in 1000 mg/kg EP group. EP has been shown to have significant free radical scavenging potential (33) and its benefits on various problems such as blood parameters in diabetic rats (34), cancer cells viability, and inflammatory factors were investigated; however, as mentioned in the introduction section, only one study showed EP benefits in gonadotoxicity.

The testosterone creation is a complicated pathway that relies on the balances between FSH, LH, GnRH, and vital factors necessary for activating Leydig cells and, eventually, spermatogenesis (35). In the meantime, ROS and oxidative stress link to steroidogenic enzyme decline, such as 17- and 3-hydroxysteroid dehydrogenase enzymes, which leads to a reduction in testosterone and LH synthesis (36, 37). In our study, we had a significant reduction of LH, FSH, and testosterone in groups treated only with CP, while this outcome is supported by the fact we had mentioned before. In the previous study, EP had a significant increase in serum and tissue hormonal levels and had activated the GnRH signaling pathway (23).

CP-induced ROS disrupts spermatogenesis through the testicular-endocrine pathway, altering the synthesis of FSH and LH, resulting in low testosterone levels (7, 38). The influence of dietary antioxidant agents in oxidative situations is discussed in many studies (39-41). A study by Ebokaiwe et al., evaluated the effect of quercetin, with antioxidant features, on CP-induced gonadotoxicity. This study revealed a significant increase in the activities of tryptophan 2, 3-dioxygenase, indoleamine 2, 3-dioxygenases, an elevated interferon-γ, interleukin 6, and testis MDA and quercetin co-administration significantly preventing their elevation (39).

In another study, the protective potential of Irbesartan against CP-induced gonadotoxicity was discussed. This study showed that CP treatment raised MDA, caspase-1, and interleukin 18 levels, as well as NF-B, NLRP3, B-cell lymphoma-2 associated X protein, caspase-3, and MT staining in testicular tissue compared to the normal control group. In comparison to the normal control group, there was a decrease in GSH, superoxide dismutase, PPAR expression, B-cell lymphoma-2 staining, circulating testosterone, and the quantity and quality of epididymal sperm. However, using Irbesartan with anti-inflammatory, anti-apoptotic, and antioxidative features regulated these altered biomarkers, and revealed the possible protective effect against testicular damage (42).

Many other studies had proved the CP-induced gonadotoxicity and the benefit of herbs, with antioxidative properties on this (43, 44). These facts support our experiment's results that CP has a potent feature in the diminution of testosterone in plasma and regulating the testicular and gamete cells.

Investigating the effect of hydroalcoholic extract of EP on sperm parameters, such as viability and sperm DNA integrity can increase our knowledge about this plant. It also suggests that further studies should be conducted on rats that have been exposed to chemotherapy.

## 5. Conclusion

As a result, leaf hydroalcoholic extract of EP pachyclada can be utilized to mitigate the adverse effects of CP, especially in higher doses. The EP extracts' therapeutic effects are based on its antioxidative function and resilience against the ROS status and oxidative stress in CP toxicity that diminishes steroidogenesis, testosterone production, and sperm functional parameters.

##  Conflict of Interest

The authors declare that there is no conflict of interest.
